# Prevalence and Genotypic Characterization of Extended-Spectrum Beta-Lactamases Produced by Gram Negative Bacilli at a Tertiary Care Hospital in Rural South Western Uganda

**DOI:** 10.9734/BMRJ/2014/9792

**Published:** 2014-08-22

**Authors:** Acaku Moses, Freddie Bwanga, Yap Boum, Joel Bazira

**Affiliations:** 1 Department of Microbiology, Mbarara University of Science and Technology, Mbarara, Uganda; 2 Department of Medical Microbiology, Makerere University College of Health sciences, Uganda; 3 MBN Clinical Laboratories Kampala, Uganda; 4 Epicenter Mbarara Research Base, Mbarara, Uganda

**Keywords:** Antibiotics, resistance patterns, sensitivity patterns, ESBL, Uganda

## Abstract

**Aim:**

To determine the prevalence and genotypic characterisation of extended spectrum beta-lactamases produced by gram negative bacilli isolated at Mbarara Regional Referral Hospital (MRRH).

**Samples:**

Gram negative clinical isolates.

**Study Design:**

Laboratory-based descriptive cross-sectional study.

**Place and Duration of the Study:**

MRRH, June and August 2012.

**Methods:**

Gram negative clinical isolates were sub cultured, and identified using biochemical tests. They were screened for ESBL by using oxyimino-cephalosporins and confirmed by double disc synergy Genotyping was performed using the PCR for TEM, SHV and CTX-M. Susceptibility pattern for the extended spectrum beta-lactamases, (ESBL) - positive isolates to other antibiotic classes was performed by the Kirby Bauer Technique.

**Results:**

A total of 484 isolates were included in the study. The commonest ESBL producers were *Escherichia coli* (34%), followed by unidentified *coliforms* (19.3%) *and Klebsiella* spp. (12.7%). Phenotypically, 88/484 were ESBL producers while genotypically 213/ 484 possessed ESBL genes. The ESBL genes were *bla*_CTX-M_ (146; 70%), *bla*_SHV_ (72; 34%) and *bla*_TEM_ (100; 47%). 87of 213 isolates expressed more than one ESBL gene. Of these 36 (7.4%) produced *bla*_CTX-M_/*bla*_SHV,_ 28 (5.8%) *bla*CTX-M /*bla*TEM, 4 (0.8%) *bla*SHV/ *bla*TEM and 19 (3.9%) *bla*CTX-M/*bla*SHV*/bla*TEM. Sixty two (16%) were phenotypically and genotypically positive, 12 (3%) of the isolates were phenotypically positive but genotypically negative and 140 (37%) isolates were phenotypically negative but genotypically positive. The ESBL producers were highly susceptible to imipenem (95%), nitrofurantoin (66%) but less susceptible to ampicillin (4%) and ticarcillin (7%).

**Conclusion:**

ESBL production among the Gram-negative clinical isolates at MRRH is very high with several isolates possessing multiple genes. The ESBL producers are highly susceptible to imipenem, but very resistant to ciprofloxacin.

## 1. INTRODUCTION

Resistance to β-lactam antimicrobial agents is on the rise worldwide. Extended-spectrum β-lactamases (ESBLs) are clinically important because they destroy cephalosporins, workhorse hospital antibiotics that are given as first-line agents to many severely-ill patients, including those with intra-abdominal infections, community acquired pneumonias and bacteraemias [1]. Delayed recognition of severe infections caused by ESBL producers, and inappropriate treatment with cephalosporins has been associated with increased mortality and morbidity. [2–4]. ESBLs are capable of hydrolyzing broad spectrum cephalosporins and monobactams.

In addition, ESBL-producing organisms exhibit co-resistance to many other classes of antibiotics resulting in limitation of therapeutic options. These enzymes can be chromosomal or plasmid-mediated and are encoded by genes such as TEM, SHV, CTX-M, VEB, PER, and OX [5].

Many ESBL producers are multi-resistant to non β-lactam antibiotics such as quinolones, aminoglycosides, chloromphenical and sulfamethoxazole-trimethoprim, narrowing treatment options [2,6,7]. Some producers achieve outbreak status, spreading among patients and locales, perhaps owing to particular pathogenicity traits.

The study was carried out to determine the prevalence and molecular characterization of Extended Spectrum Beta-Lactamases (ESBLs) produced by Gram-negative bacilli isolated at Mbarara Regional Referral Hospital (MRRH).

## 2. MATERIALS AND METHODS

This was a laboratory-based descriptive cross-sectional study that was conducted in the Microbiology Department of Mbarara University of Science and Technology. The study included all Gram-negative bacilli isolated from clinical samples (blood culture, urine, stool, peritoneal fluid, pus swab, high vaginal swab, cerebrospinal fluid, pleural fluid/aspirate, sputum, urethral and nasal swabs) collected from in patient and out patients of Mbarara Regional Referral Hospital between June and August 2012.

### 2.1 Culture and Identification of the Isolates

The specimens were cultured on Blood agar, Chocolate agar, Cystiene Lactose Electrolyte Deficient agar, (CLED), XLD and MacConkey agar depending on the sample type and incubated at 37°C over night. Identification of the isolates was carried out using conventional biochemical tests [8,9].

### 2.2 Phenotypic Screening for ESBLs

Screening for reduced susceptibility to third generation cephalosporins was carried out using cefotaxime and ceftazidime discs and double-disk synergy (DDS) method. The antibiotics used were; *ceftazidime (*30μg) and ceftazidime-clavulanic acid (20+10μg), *cefotaxime (*30μg) and cefotaxime-clavulanic acid (20+10μg). These antibiotics were placed at a distance of 30mm from each other. The plates were incubated overnight at 37°C. An increase of at east 5mm in the zone diameter for ceftazidime-clavulanic acid versus the zone diameter with ceftazidime tested alone was used to confirm the presence of ESBLs. A similar interpretation criterion was used when cefotaxime-clavulanic acid versus cefotaxime alone was uses, as recommended by the Clinical and Laboratory Standards Institute [10].

### 2.3 Molecular assays for detection of the *β*-Lactamase Genes

All the polymerase chain reaction (PCR) assays were performed at MBN Clinical Laboratories, Kampala Uganda where all the isolates were tested for the ESBL-resistance genes TEM, SHV and CTX-M, using primers as published earlier [11–13].

Frozen isolates were thawed and subcultured in *Luria*-*Bertani* and then incubated at 35 (+/−2) for 16–18 hrs.

#### 2.3.1 DNA extractiion

2ml of the overnight culture was centrifuged at maximum speed of 13,000xg for 2 minutes, the supernatant fluid removed and the deposit re-suspended in 500 μl of TE buffer. This was spun again as before and the supernatant removed by gentle aspiration. To the deposit was added 100 μl of PCR water, vortexed, boiled for 15 minutes, cooled and centrifuged to collect the supernatant. This was then used as a DNA template for amplification of SHV, TEM, and CTX-M beta-lactamase genes. The extracted DNA from bacterial isolates was used as a template to detect SHV, TEM, and CTX-M beta-lactamase genes.

#### 2.3.2 Amplification

PCR was carried out in a solution containing 200 μM concentration of dNTPs, 10 pM of each primer, 0.8mM/μl MgCl_2_, 0.5 U *Taq* polymerase and 50 ng DNA template in a final volume of 25 μl. The following thermocycler program was carried out for PCR experiments: 4 minutes denaturation at 94°C followed by 32 cycles of 1 minute at 94°C, 1 minute at the annealing temperature (55°C for *blaSHV* and *blaCTX-M* and 58°C for *blaTEM*) and 1 minute at 72°C with a final extension period of 10 minute at 72°C. *K. pneumoniae* 7881 *and E. coli* ATCC 35218 containing *bla*SHV, *bla*CTX-M and *bla*TEM gene were used as controls. 10 X master mix from AB gene was prepared (stable for one year at −20°C).

##### 2.3.1.1 Gel electrophoresis

All PCR amplicons were verified by gel electrophoresis for amplicons of the following sizes (blaTEM 859bp, blaSHV 865bp, and blaCTX-M 544bp) which was performed at a voltage of 120V for 1 hour (Fig. 2).

### 2.4 Resistance Patterns of ESBL-Positive Isolates

The susceptibility pattern of the confirmed ESBL-positive isolates was performed for the following antibiotics: gentamicin (10μg), ampicillin (10μγ), nitrofurantoin (300μg), ciprofloxacin (5μg), chloromphenicol (30μg), septrin (1.25/23.75μg), ticarcillin (75μg), imipenem (10μg) and nalidixic acid (30μg). The results were expressed as susceptible or resistant according to the criteria recommended by the Clinical Laboratory Standards Institute (CLSI) [10].

### 2.5 Quality Control

*Klebsiella pneumoniae* ATCC 700603 and *Escherichia coli* ATCC 25922 were used for the quality control of testing methods.

### 2.6 Data Analysis

Data was entered using EPI info version 7, exported to Excel and Stata version 11 (Stata Corporation, College Station, TX, USA) statistical packages for analysis.

## 3. RESULTS

**Fig. 1:**
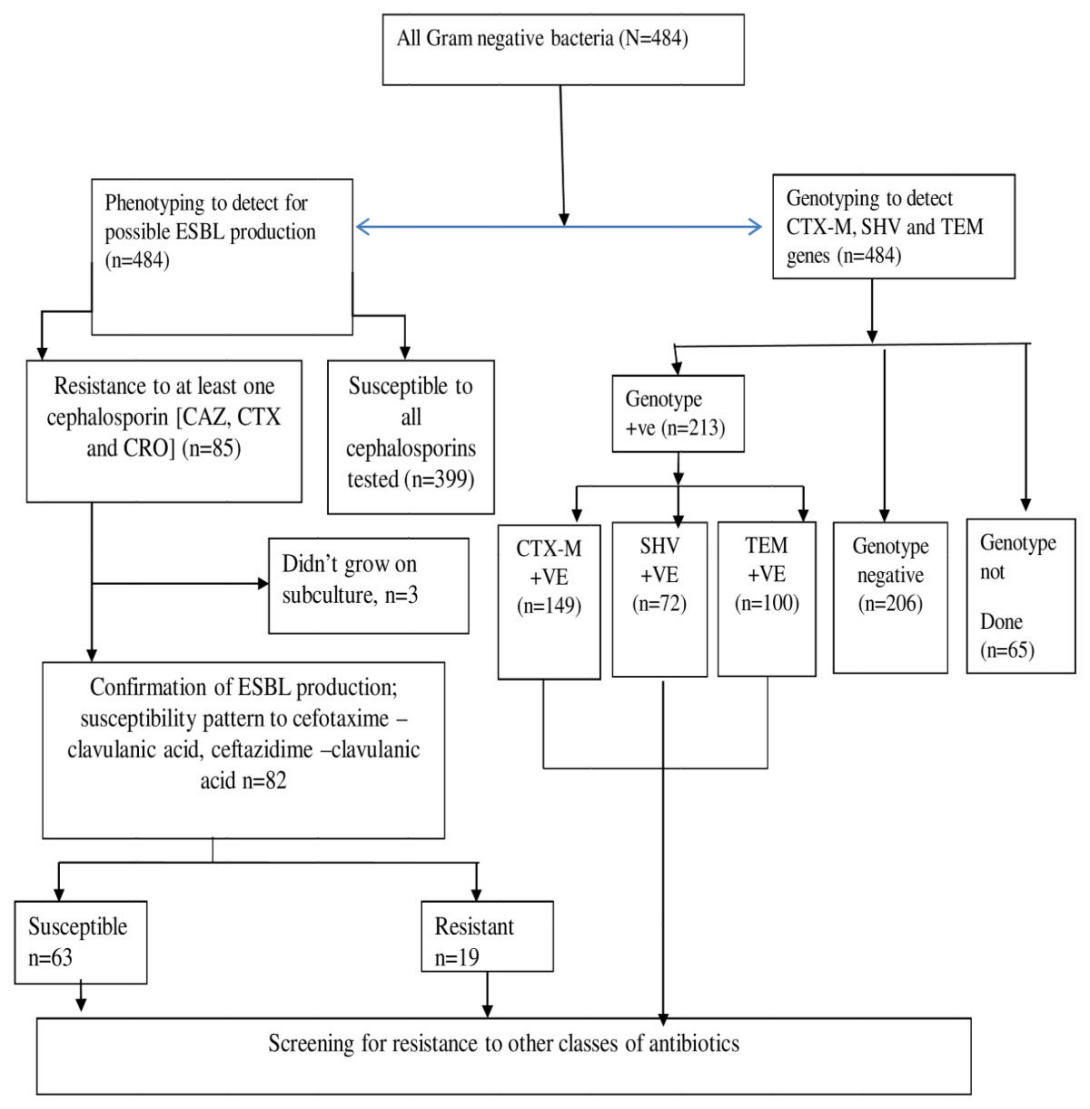
Experimental design

**Fig. 2:**
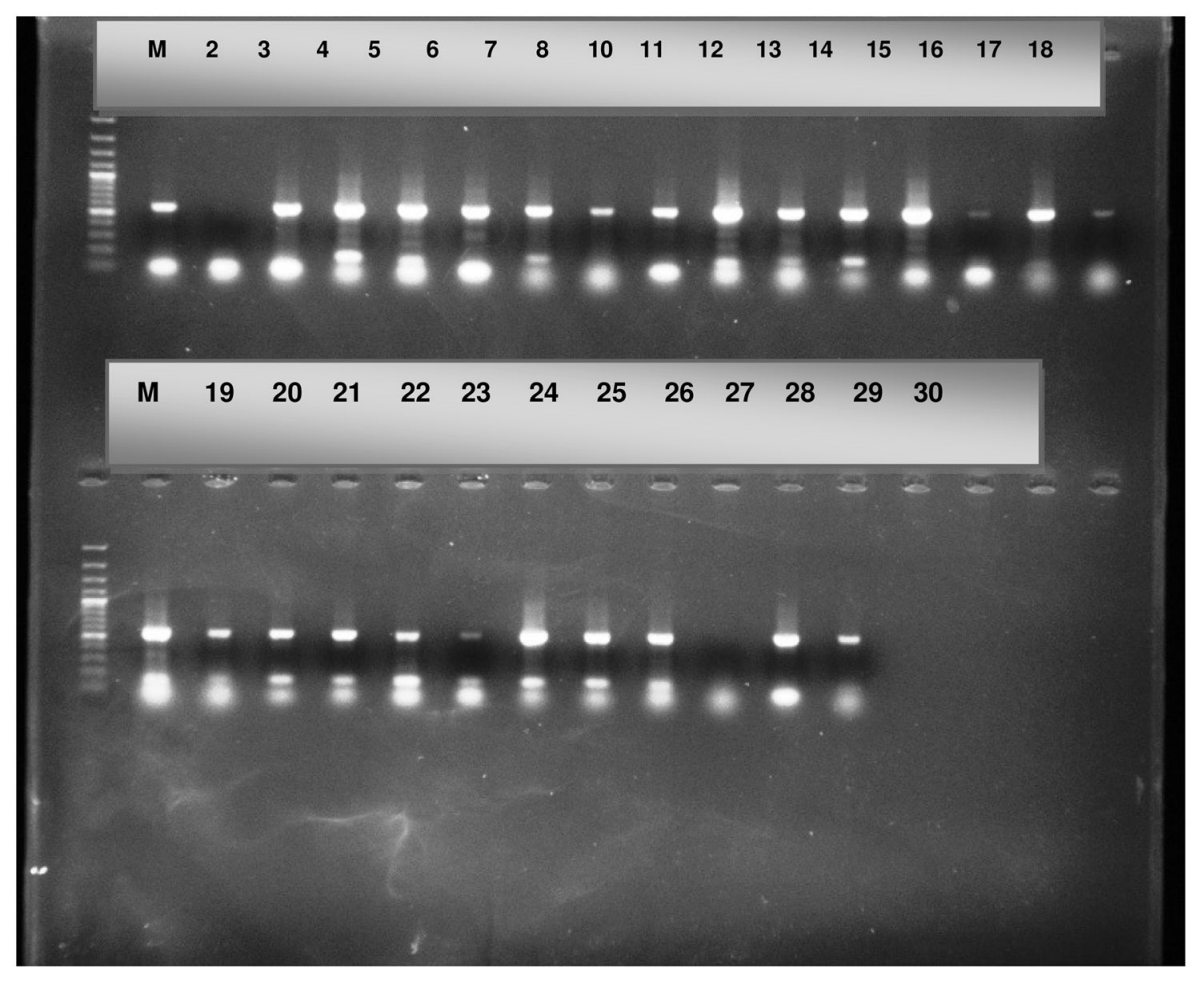
Amplified PCR products

**Fig. 3:**
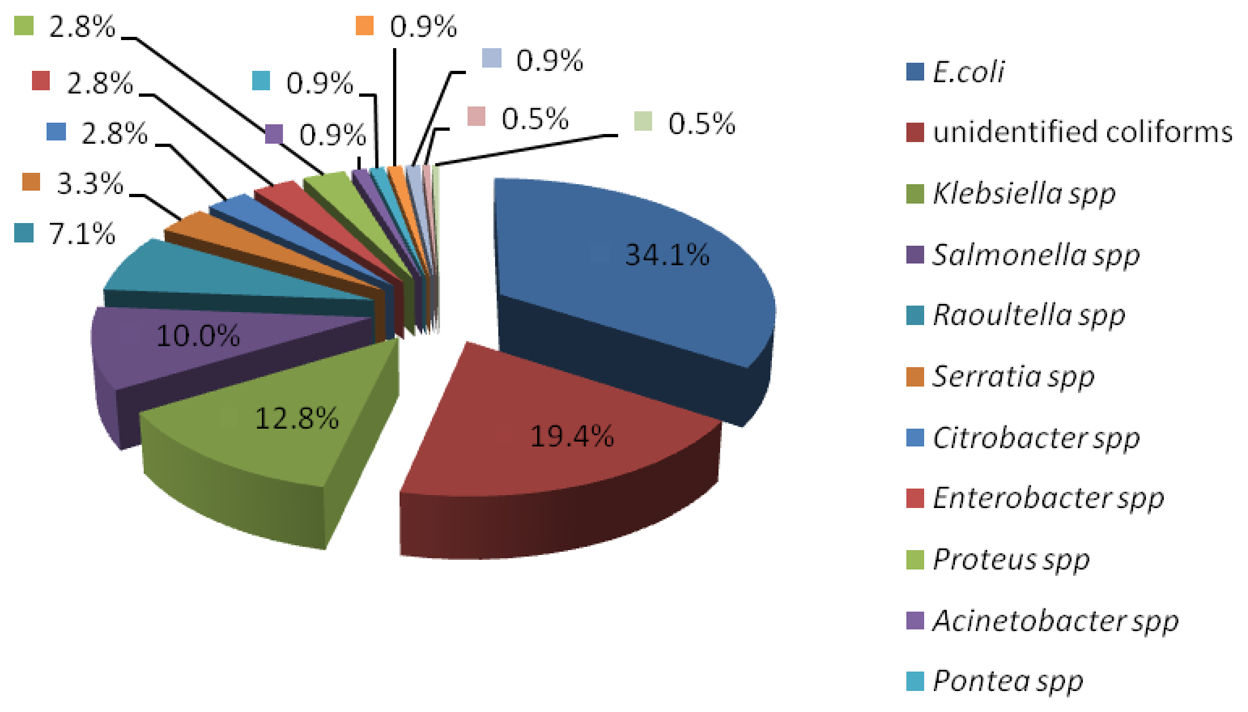
ESBL-producing bacteria

Of the 484 isolates phenotypically screened, 85 (29.9 %) isolates were resistant to at least one cephalosporin (ceftazidime, cefotaxime or ceftriaxone). Three of these isolates did not grow, and on phenotypic confirmation, 19 (23.2%) of the 82 isolates were confirmed to be ESBL (Fig. 1). The most common ESBL producing bacterial isolate was *Escherichia coli (34.0%), Klebsiella* spp. *(12.7%) and Salmonella spp* (9.9 %) (Fig. 3). Of the 419 isolates that were genotyped, 321 (50.8 %) isolates carried an ESBL coding gene. Majority of the isolates carried the *bla*_CTX-M_ (146/213), followed by the blaTEM (100/213) and blaSHV (72/213).

As shown in Table 1, eighty seven, 87 (40.8 %) out of the 213 isolates produced more than one ESBL gene genes. Majority of these isolates produced both CTX-M and SHV.

Gel electrophoresis displaying the amplified blaSHV (862 bp) genes. Lane 2 positive control and Lane 3 negative control. Lanes 4–27, 29 and 30 are positive, Lane 28 is the only negative one. Lanes M represent the molecular weight marker (50 bp DNA ladder, Promega, Madison, USA).

**Fig. 4:**
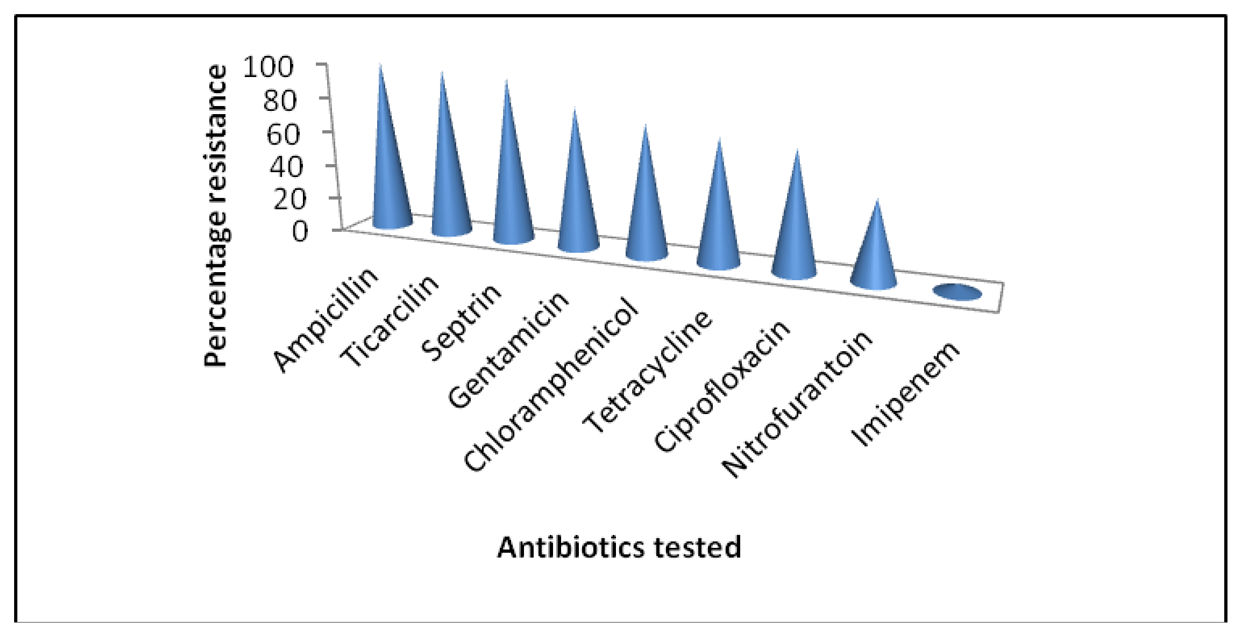
Resistance pattern of ESBL producing organisms

As shown in Fig. 4, the ESBL producers were highly resistant to ticarcillin, ampicilin, tetracycline, and chloramphenicol but were highly susceptibility to imipenem.

## 4. DISCUSSION

Using genotypic methods, the prevalence of ESBL in this study, was about 51 %. This correlates to what has been reported in other studies such as that from Israel (47 %) [14], and Nigeria (36.6 %)[15], but is much higher than that reported from Italy (6.3 %), Greece (27.4 %), Netherlands (2.0 %) and Germany (2.6%) [16]. The high prevalence has been majorly reported in low and middle income countries. This implies that the prevalence of ESBL may have a relationship with the economic status of a society. It may also have to do with the easy access to antibiotics in the study area. In our study, the commonest ESBL gene was CTX-M. This is different from studies performed elsewhere, where the commonest gene is SHV [17]. Furthermore, 87 out of the 213 isolates were able to express more than one resistance gene; *bla*_CTX-M_/ *bla*_SHV,_ *bla*_CTX-M_ /*bla*_TEM_, *bla*_SHV_/ *bla*_TEM_ and *bla*_CTX-M/_ *bla*_SHV_*/bla*_TEM_. This is in agreement with studies carried out in other parts of the world that one bacterial isolate can express more than one resistance gene for example in Turkey, 68 had SHV and TEM, 61 (55.9 %) TEM and CTX-M, and 54 (49.5 %) SHV and CTX-M genes [17] and also in Iran, 7 isolates (21.87%) contained both genes TEM and SHV[18].

In this study, the predominant ESBL producers were *Escherichia coli,* unidentified coliform and *Klebsiella spp.* These findings are similar to previous studies reported elsewhere [19–23]. The susceptibility pattern of the ESBL-producing isolates in this study indicates a cross resistance of these ESBLs to many other common antibiotics. This has been recorded in other studies [6,7,24–27]. The mechanism behind this multiresistance phenomenon is genetic; it may be chromosomal or plasmid mediated. The gene encoding for resistance to ESBL and other antibiotic classes (e.g. quinolones) are often located on the same mobile DNA element (plasmid) [28], thus propagation of this plasmid during conjugation leads to development of multidrug resistance in previously sensitive organisms.

There was a discrepancy between genotypic and phenotypic methods for the detection of ESBL. This is due to the fact that genotypic assays are basically used to show the presence or absence of a defined resistance determinant gene. This equally is supported by the fact that the low prevalence phenotypically could possibly be due to non-expression of the genes. However low-level resistance mechanisms can be detected genotypically which otherwise can be difficult to detect using phenotypic methods because this is dependent on gene expression. Thus, if the genes are not expressed, then detection becomes difficult to achieve.

## 5. CONCLUSION

The prevalence of ESBL in our study is high, with the commonest ESBL gene being CTX-M, and a high proportion of isolates containing multiple genes. These ESBL producing isolates are highly resistant to ampicilin, ticarcilin, tetracycline and chloramphenicol are highly susceptible to imipenem.

## Figures and Tables

**Table 1:** Combinations of ESBL genes produced by the isolates

Genotype	Frequency	Percentage	Cum. freq
CTX-M and SHV (n=87)	36	41.4	41.4
CTX-M and TEM (n=87)	28	32.2	73.6
SHV and TEM (n=87)	04	4.6	78.2
CTX-M, SHV and TEM (n=87)	19	21.8	100
